# High CD8/CD33 ratio in peritoneal metastatic lesions is associated with favorable prognosis in gastric cancer

**DOI:** 10.1002/cnr2.1389

**Published:** 2021-04-01

**Authors:** Takahisa Yamaguchi, Jun Kinoshita, Hiroto Saito, Mari Shimada, Siro Terai, Hideki Moriyama, Koichi Okamoto, Isamu Makino, Keishi Nakamura, Hidehiro Tajima, Itasu Ninomiya, Sachio Fushida

**Affiliations:** ^1^ Department of Gastroenterological Surgery, Division of Cancer Medicine Kanazawa University Graduate School of Medical Science Kanazawa Japan

**Keywords:** CD8^+^ lymphocytes, gastric cancer, MDSCs, peritoneal metastasis, TILs

## Abstract

**Background:**

Tumor‐infiltrating lymphocytes (TILs) and other immune cells have been reported as a prognostic factor in several tumors, including gastric cancer, and they play an important role in antitumor effect at the primary site. There were few reports on the immune status in peritoneal metastatic lesions for gastric cancer.

**Aims:**

The aims of this study were to assess the prognostic significance of TILs (CD4, CD8, CD19, regulatory T cells [Tregs]), and myeloid‐derived suppressor cells (MDSCs) in peritoneal metastatic lesions.

**Methods:**

We retrospectively investigated 60 patients for gastric cancer with peritoneal metastasis who were treated between 2009 and 2016 in our institute. Immunohistochemistry for CD4, CD8, CD19, FOXP3, and CD33 was performed in the peritoneal metastatic lesions. The absolute numbers of immune cells and ratios were evaluated, and the relationship between immune‐related marker and overall survival (OS) was investigated.

**Results:**

A high infiltration of CD8^+^ lymphocytes or high CD8/CD33 ratio was a better prognosis for OS in univariate analysis using all immunologic variables (*P* = .012, *P* = .001). In multivariate analysis for clinical and immunologic variables, high CD8/CD33 ratio was identified as an independent prognostic factor for OS (Hazard ratio: 0.291, 95% confidence interval: 0.126‐0.670, *P* = .004).

**Conclusion:**

High CD8/CD33 ratio and high infiltration of CD8^+^ lymphocytes in peritoneal metastatic lesions were favorable prognoses for gastric cancer patients with peritoneal metastasis. It is necessary to modify the immune microenvironment result to increase the level of CD8^+^ lymphocytes in the peritoneal metastatic lesions.

**Abbreviations**
CTLs: cytotoxic T lymphocytes
MDSCs: myeloid‐derived suppressor cells
OS: overall survivals
TAMs: tumor‐associated macrophages
TGF‐β: transforming growth factor‐β
TILs: Tumor‐infiltrating lymphocytes
Tregs: regulatory T cells
VEGF: vascular endothelial growth factor

## 1 BACKGROUND

Globally, gastric cancer is one of the most life‐threatening diseases in the world, and a major cause of cancer‐related deaths.^1^, ^2^ Peritoneal metastasis is an incurable disease that occurs frequently in recurrent gastric cancer and associates with poor prognosis. Importantly, molecular mechanisms of peritoneal metastasis are poorly understood. Although various treatments have been developed for peritoneal metastasis, including systemic chemotherapy, intraperitoneal chemotherapy, sufficiently satisfactory outcomes have yet to be achieved^3^, ^4^, ^5^; this indicates the need for novel treatment strategies, in addition to conventional surgery and chemotherapy.

It is thought that the progression and suppression of tumor depend on various cell‐cell interactions in local and metastatic lesions in the tumor microenvironment. Several studies have attempted to investigate the relationship between tumor‐infiltrating lymphocytes (TILs) and prognosis in several cancers, suggesting that TILs accumulation was associated with better prognosis.^6^, ^7^ CD8 is a surface antigen expressed in cytotoxic T lymphocytes (CTLs) that recognize specific antigen and eliminate the foreign objects. Several studies have reported that high infiltration of CD8^+^ lymphocytes was associated with better overall survival (OS) and disease‐free survival (DFS) in gastric cancer.^8^, ^9^, ^10^ Furthermore, CD4^+^ helper T cells play an important role in adaptive immune system by releasing cytokines.^11^ Higher infiltration of CD4^+^ lymphocytes is also related to favorable prognosis in gastric cancer.^12^ Furthermore, MDSCs have been shown to contribute to cancer evasion by suppressing the T lymphocytes and natural killer cells in tumor microenvironment. MDSCs commonly express the cell surface marker CD33 and CD11b, and CD33 positive cells in advanced gastric cancer correlated with worse prognosis.^13^

Though many studies have been progressing in the primary lesions for immune microenvironment, there were few reports on the immune status in metastatic lesions, including peritoneal metastasis. We have previously reported that tumor‐associated macrophages (TAMs) and mast cells contributed to the tumor progression during peritoneal metastasis.^14^, ^15^ However, the role of lymphocytes in peritoneal metastasis is not well understood. It is important to evaluate the tumor immune microenvironment to develop new treatment strategies. In this study, we investigated the expression of immunocompetent cells and the relationship between cell density and prognosis in gastric cancer with peritoneal metastasis.

## 2 MATERIALS AND METHODS

### 2.1 Patients and specimens

Sixty patients for gastric cancer with peritoneal metastasis who were treated in our institute in the period between 2009 and 2016 were investigated. Prior to chemotherapy, the specimens from peritoneal metastases were collected either during surgery or staging laparoscopy. Age, sex, histologic type (according to Lauren classification), TNM stage (according to TNM classification), and European Cooperative Oncology Group (ECOG) performance status were evaluated by reviewing medical records. WHO Classification of Tumours, 5th edition^16^ was used for classification of cancer related factors. Peritoneal metastasis was categorized into three classifications (P1a‐P1c) according to the 15th edition of the General Rules for Gastric Cancer Study of the Japanese Research Society for Gastric Cancer. The ascites level was classified into four groups (none, mild, moderate, and severe) using computed tomography as we previously reported.^17^ Patients received the chemotherapy of physician's choice including taxane‐based intraperitoneal chemotherapy.^4^ Immune checkpoint inhibitor was not used in all cases. Among the patients who were judged as resectable, gastrectomy was performed 4 to 8 weeks after their last day of chemotherapy.

### 2.2 Immunohistochemistry

The specimens were dewaxed in xylene and rehydrated through graded ethanol for immunohistochemistry. Endogenous peroxidase activity was blocked by incubating in 3% H_2_O_2_ in methanol for 20 minutes at room temperature. Antigen retrieval was done using microwaving sections in citrate buffer at 95°C for 10 minutes. Subsequently, the sections were incubated for 2 hours at room temperature with primary antibody, anti‐CD4 antibody (1:100, anti‐CD4 mouse monoclonal, 4B12; Nichirei, Tokyo, Japan), anti‐CD8 antibody (1:200, anti‐CD8 rabbit polyclonal, ab4055; Abcam, Tokyo, Japan), anti‐FOXP3 antibody (1:50, anti‐FOXP3 mouse monoclonal, 236A/E7; Abcam), anti‐CD33 antibody (1:100, anti‐CD33 mouse monoclonal antibody, NCL‐L‐CD33; Leica Biosystems, NewCastle upon Tyne, UK), and anti‐CD19 antibody (1:250, anti‐CD19 rabbit monoclonal, EPR5906; Abcam, Tokyo, Japan). Secondary antibody was subsequently performed for 1 hour, and then the sections were developed in DAB solution.

### 2.3 Evaluation of immunostaining

To evaluate the expression of immune‐related markers in the specimens, five non‐overlapping intratumoral fields were counted under high power fields. All slides were counted by two researchers (TY and JK). The mean numbers of positive cells in five fields were calculated in each antibody. The median number was used to divide the patients into two groups (low or high).

### 2.4 Statistical analysis

We investigated the differences among the data sets using Fisher's exact test using the computer software package SPSS version 25 (SPSS, Chicago, IL, USA). Survival curves were created using the Kaplan‐Meier method and the log‐rank test. OS was calculated from the date the diagnosis of peritoneal metastasis was established to death from any cause or the latest follow‐up. The influence of each factor on patients’ survival was evaluated using Cox regression analysis. Multivariate analysis of survival distributions was performed using Cox proportional hazards regression models. *P* values < .05 were considered a statistically significant difference.

## 3 RESULTS

### 3.1 Patient clinicopathological characteristics

The characteristics of 60 patients are shown in Table 1. The median age of the patients was 63 years (28‐83 years). Twenty‐five patients were male (41.7%) and 48 (80.0%) had initial treatment. Thirty‐nine patients had a history of gastrectomy and 21 had not undergone gastrectomy. Four patients (6.7%) were performance status (PS) ≥2, and 56 (93.3%) were PS of either 0 or 1. P status was P1a in 8 (13.3%), P1b in 4 (6.7%), and P1c in 48 (80.0%) patients. The ascites levels were as follows: 23 (38.3%) were none, 17 (28.3%) were mild, 7 (11.6%) were moderate, and 13 (21.7%) patients were severe.

**TABLE 1:** Clinical and pathological data of 60 patients with gastric cancer with peritoneal metastasis

Characteristics		(n = 60)
Age, years; median (range)		63 (28‐83)
Gender	Male	25
	female	35
Initial or Recurrence	Initial	48
	Recurrence	12
gastrectomy	−	21
	+	39
ECOG performance status	≧0,1	56
	2	4
Borrman macroscopic type	1	1
	2	3
	3	25
	4	26
	5	5
Histology (Lauren classification)	Intestinal	12
	diffuse	48
P status	P1a	8
	P1b	4
	P1c	48
Ascites	None	23
	Mild	17
	Moderate	7
	Severe	13
Other distant metastasis	−	50
	+	10

*Note*: P1a, greater omentum, lesser omentum anterior lobe of the transverse colonic membrane, or membrane of the pancreatic surface or spleen. P1b, a few scattered metastases to upper abdominal peritoneum. P1c, many metastases to the middle or lower peritoneum.

Abbreviation: ECOG, European Cooperative Oncology Group.

### 3.2 Relationship between clinical variables and OS

In univariate analysis of clinical variables, age, gender, initial or recurrence, ECOG performance status, Borrmann type, and pathology P status were not associated with OS (Table 2). On the other hand, OS was significantly lower in patients with gastrectomy (−), severe ascites, and presence of distant metastases in addition to peritoneal metastasis.

**TABLE 2:** Relationship between patient characteristics and overall survival

Variable		OR	95%CI	Number of patients	*P* value
Age, years	≧70	1.188	0.648‐2.108	16	.313
	<70			44	
Gender	Male	0.97	0.322‐2.920	26	.956
	Female			34	
Initial or Recurrence	Initial	1.088	0.502‐2.359	48	.831
	Recurrence			12	
Gastrectomy	−	0.48	0.243‐0.952	21	.036
	+			39	
ECOG performance status	0,1	2.885	0.378‐12.457	56	.309
	≧2			4	
Borrman type	Type4	0.982	0.538‐1.791	27	.952
	Not			33	
Histology (Lauren classification)	Intestinal	0.743	0.379‐1.456	12	.385
	Diffuse			48	
P status	1a,1b	2.044	0.969‐4.312	13	.056
	1c			47	
Ascites	None‐Moderate	2.217	1.101‐4.467	46	.022
	Severe			14	
Other distant metastasis	−	3.023	1.351‐6.764	9	.005
	+			51	

Abbreviation: ECOG, European Cooperative Oncology Group.

### 3.3 Evaluation of immune‐related cells in peritoneal metastasis

**FIGURE 1:**
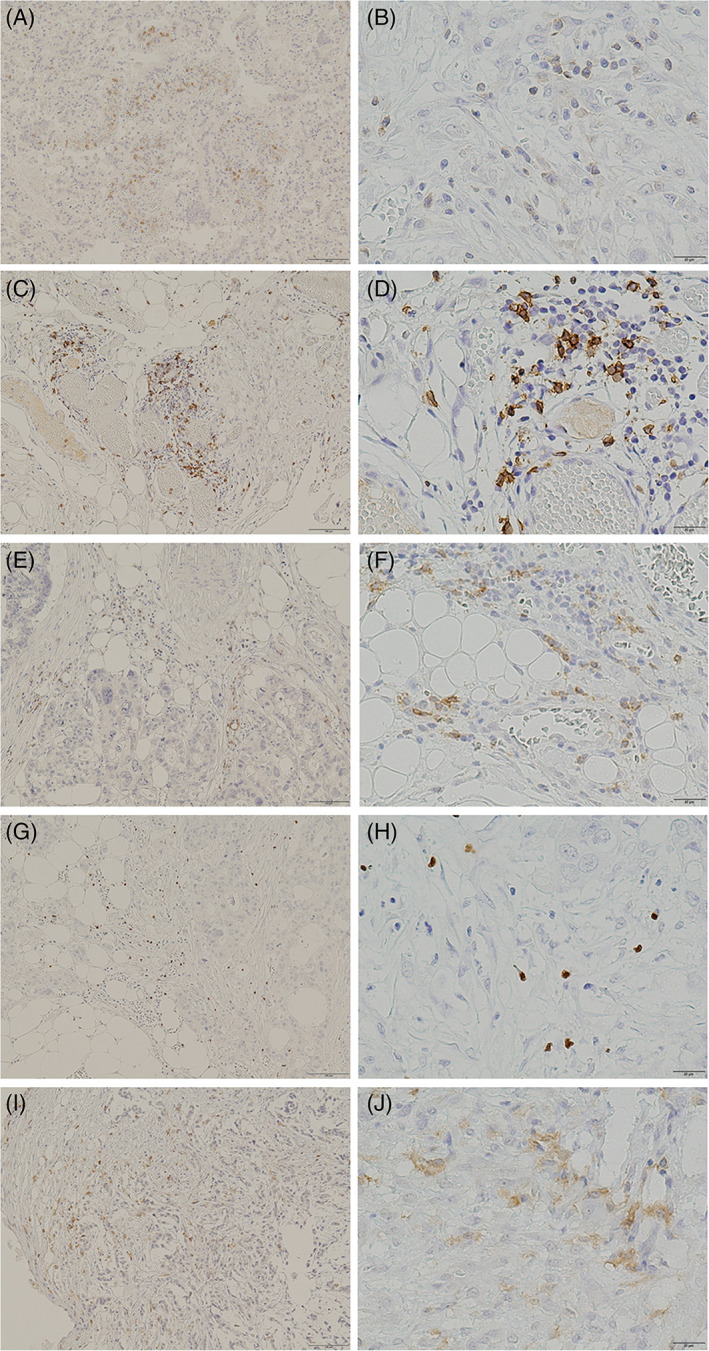
Representative immunostainings of peritoneal metastatic specimens from gastric cancer patients. A, CD4 (helper T lymphocytes, original magnification ×100). B, CD4 (original magnification ×400). C, CD8 (cytotoxic T lymphocytes [CTLs], original magnification ×100). D, CD8 (original magnification ×400). E, CD19 (B lymphocytes, original magnification ×100). F, CD19 (original magnification ×400). G, Foxp3 (regulatory T lymphocytes, original magnification ×100). H, Foxp3 (original magnification ×400). I, CD33 (myeloid‐derived suppressor cells [MDSCs], original magnification ×100). J, CD33 (original magnification ×400)

We investigated the five immunologic parameters (CD4 as helper T cells, CD8 as cytotoxic T cells, CD19 as B cells, FOXP3 as regulatory T cells [Tregs], and CD33 as myeloid‐derived suppressor cells [MDSCs]). CD4, CD8, CD19, and CD33‐positive cells showed cell membrane staining, whereas FOXP3‐positive cells exhibited nuclear staining (Figure 1A‐J).

The median number of cells positive for CD4, CD8, CD19, FOXP3, and CD33 were 6.1, 9.3, 3.6, 3.9, and 10.4. Based on the median numbers, all patients were divided into low or high density groups in each antibody, and then we performed the Kaplan‐Meier test, followed by univariate and multivariate analyses.

### 3.4 Survival according to immunological parameters

**FIGURE 2:**
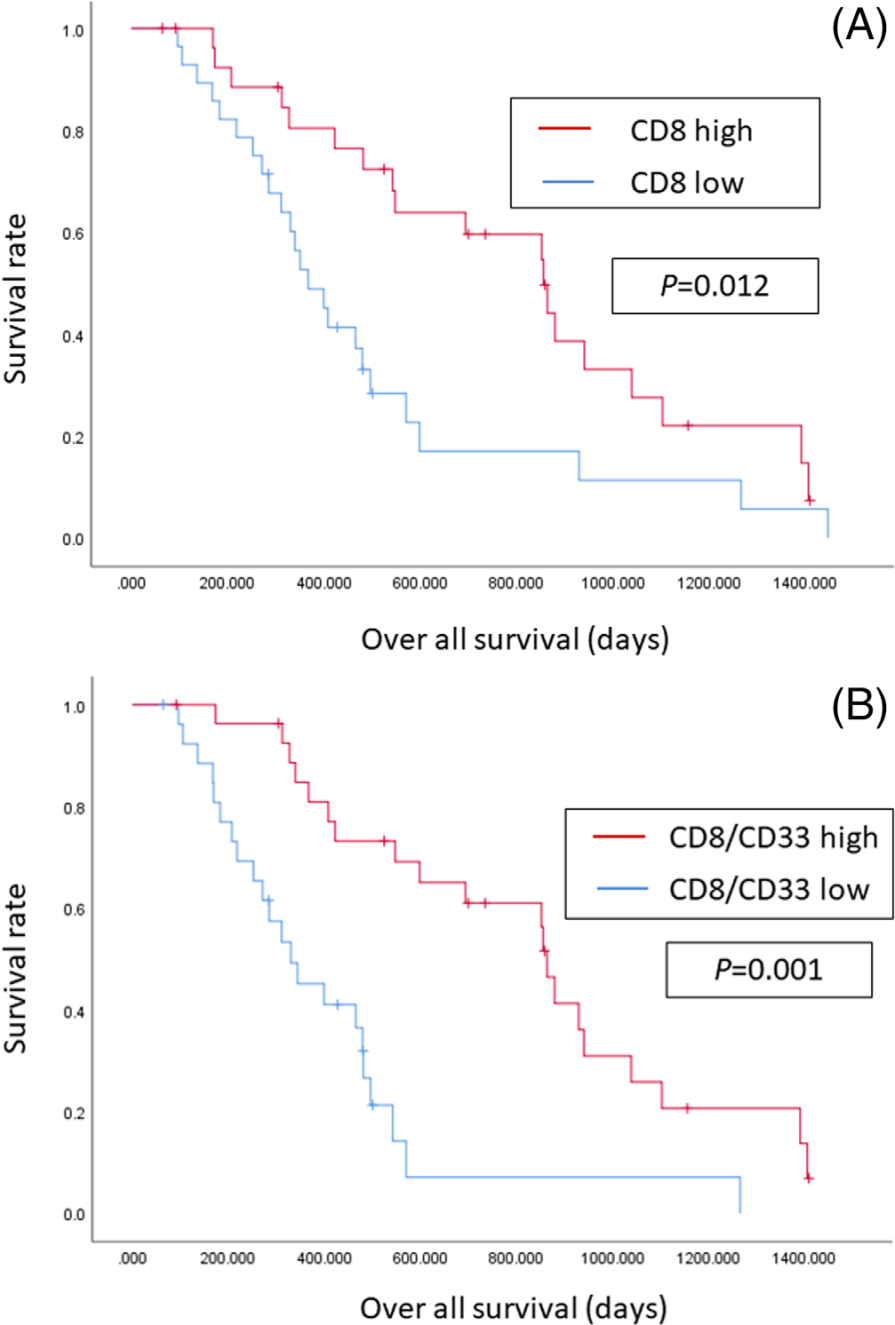
Survival curves in 60 patients according to the density of CD8^+^ TILs and CD8/CD33 ratio. A, The prognosis in patients with CD8 high‐density groups was significantly better than that in those with CD8 low‐density (*P* = .012, log‐rank). B, The prognosis in patients with high CD8/CD33 ratio was significantly better than that in those with low CD8/CD33 ratio (*P* = .001, log‐rank)

As shown in Table 3, univariate analysis indicated the CD8 high‐density groups were associated with better prognosis (*P* = .012; hazard ratio [HR]: 0.457 95% confidence interval [CI]: 0.245‐0.855). Median survival time (MST) in CD8 low density groups was 12.2 months, while that in CD8 high density groups was 28.5 months. In contrast, the densities of CD4, FOXP3, CD19, and CD33 cells were not associated with OS. For further validation of our results, combined ratio was also calculated. The subgroup of patients with high CD8/CD33 ratio had significantly improved OS (*P* = .001; HR: 0.275; 95%CI: 0.140‐0.542). MST in low CD8/CD33 ratio groups was 11.0 months, while that in high CD8/CD33 ratio groups was 28.7 months. On the other hand, CD8/CD4, FOXP3/CD4, CD8/FOXP3, and CD4/CD33 ratios were not associated with OS. Figure 2A,B shows the Kaplan‐Meier curves in 60 patients according to CD8 expression and CD8/CD33 ratio.

**TABLE 3:** Relationship between immunological parameters and overall survival

Variable	Median		Number of patients	HR	95%CI	*P* value
CD4	6.1	Low	34	0.575	0.296‐1.118	.099
		High	26			
CD8	9.3	Low	32	0.457	0.245‐0.855	.012
		High	28			
CD19	3.6	Low	41	0.974	0.490‐1.937	.941
		High	19			
FOXP3	3.9	Low	35	0.993	0.539‐1.829	.981
		High	25			
CD33	10.4	Low	31	1.461	0.784‐2.723	.23
		High	29			
CD8/CD4	1.13	Low	29	1.027	0.552‐1.909	.933
		High	31			
FOXP3/CD4	0.41	Low	30	1.526	0.803‐2.900	.194
		High	30			
CD8/FOXP3	2.47	Low	33	0.547	0.289‐1.032	.059
		High	27			
CD8/CD33	0.92	Low	32	0.275	0.140‐0.542	.001
		High	28			
CD4/CD33	0.57	Low	30	0.562	0.289‐1.092	.085
		High	30			

Abbreviations: CI, confidence interval; FOXP3, forkhead box P3; HR, hazard ratio.

### 3.5 Multivariate analysis

Clinicopathological features and immunohistopathologic variables showing *P* ≦ .1 by univariate analysis were adopted as covariates when multivariate Cox proportional hazards analysis was performed (Table 4). Because there was an interaction between CD8 expression and CD8/CD33 ratio, multivariate analysis with Cox proportional hazards model was performed separately.

**TABLE 4:** Multivariate analysis of factors associated with prognosis in gastric cancer with peritoneal metastasis

Variable		No. of patients	HR	95%CI	*P* value
**Multivariate analysis with the density of CD8** ^**+**^ **TILs**					
Gastrectomy	−	21	0.244	0.272‐1.392	.244
	+	39			
P status	1a,1b	13	1.517	0.638‐3.604	.345
	1c	47			
Ascites	None‐Moderate	46	1.327	0.557‐3.163	.523
	Severe	14			
Other distant metastasis	−	9	2.438	0.988‐6.017	.053
	+	51			
CD4	Low	34	0.772	0.370‐1.611	.491
	High	26			
CD8	Low	32	0.564	0.283‐1.123	.103
	High	28			
**Multivariate analysis with the CD8/CD33 ratio**					
Gastrectomy	−	39	0.558	0.243‐1.280	.169
	+	21			
P status	1a,1b	13	1.762	0.699‐4.444	.23
	1c	47			
Ascites	None‐Moderate	46	0.796	0.318‐1.994	.627
	Severe	14			
Other distant metastasis	−	9	2.083	0.839‐5.171	.114
	+	51			
CD4	Low	34	0.766	0.364‐1.611	.482
	High	26			
CD8/CD33	Low	32	0.291	0.126‐0.670	.004
	High	28			

Abbreviations: CI, confidence interval; HR, hazard ratio; TILs, tumor‐infiltrating lymphocytes.

High CD8/CD33 ratio was an independent prognostic factor in the multivariate analysis for OS (*P* = .004; HR: 0.291; 95%CI: 0.126‐0.670), but the high infiltration of CD8^+^ lymphocytes did not significantly differ in multivariate analysis (*P* = .115).

## 4 DISCUSSION

The abdominal cavity is thought to be a special environment where various cells are present and involved in tumor progression and suppression. Yet immune function in peritoneal cavity has not been sufficiently identified. In this study, we demonstrated that high infiltration of CD8^+^ lymphocytes and high CD8/CD33 ratio in peritoneal metastatic lesions were favorable prognostic factors. As far as we know, this is the first report showing the relationship between TILs and OS for gastric cancer with peritoneal metastasis. We have previously reported that the number of CD8^+^ lymphocytes was significantly lower in peritoneal metastasis site than in primary site.^18^ On the other hand, the present study indicated that the prognosis was better in patients with high infiltration of CD8^+^ lymphocytes groups than in those with low infiltration of CD8^+^ groups, even in peritoneal metastasis. Kitayama et al^19^ reported that MST in patients with peritoneal metastasis who underwent multidisciplinary treatment including intraperitoneal chemotherapy was 15.1 to 24.6 months. Compared to these results, high density of CD8^+^ lymphocytes in peritoneal metastatic lesion was associated with favorable prognosis in our study. Decreased infiltration of CTLs is thought to be reflected by the immunosuppressive microenvironment, resulting in difficulties to treat patients with peritoneal metastasis.

The presence of CTLs in the tumor microenvironment induces the host immune response to tumor antigens, and inhibits tumor progression.^20^ Our results showed that high densities of TILs related to adaptive immunity contributed to chemosensitivity and favorable prognosis.

The reason for the different rate in CD8^+^ lymphocytes infiltration in peritoneal metastasis is unknown. Previously, we have established a peritoneal tumor model by co‐inoculating the mouse gastric cancer cell line YTN16 and the mouse myofibroblast cell line LmcMF into the C57BL6/J mice.^18^ A mouse model co‐inoculated with cancer cells and LmcMF, which functions as cancer‐related fibroblasts (CAFs), showed less CD8^+^ cell infiltration than cancer cells alone. These results indicated that CAFs, which are one of the major components of stroma in peritoneal metastasis, interfered with the accumulation of CD8^+^ lymphocytes by secreting several cytokines or chemokines such as vascular endothelial growth factor (VEGF) and transforming growth factor‐β (TGF‐β), which act as immunosuppressors in the tumor microenvironment. VEGF presence results in the decrease in CD8^+^ lymphocyte's proliferation and infiltration ability into tumor site.^21^, ^22^ Furthermore, TGF‐β inhibits the proliferation of CD8^+^ lymphocytes, and suppresses the ability of cytotoxicity.^23^ Indeed, increased concentration of both VEGF and TGF‐β have been reported in peritoneal metastasis,^17^, ^24^ thus CTL suppression occurred more strongly.

In this study, CD4^+^ TILs in metastatic lesions were not associated with OS. Helper T cells promote antitumor immunity by numerous mechanisms such as antigen presentation, T cell activation, and effector function. Li et al^25^ reported that CD4^+^ lymphocytes in gastric cancer were not significantly associated with survival outcomes, which were similar to our results. Meanwhile, Liu et al^26^ investigated that the high density of CD4^+^ lymphocytes resulted in improved survival. The relationship between CD4^+^ TILs in tumor site and prognosis was controversial.^27^ CD4^+^ lymphocytes include the Tregs or IL‐17 secreting Th17 cells. IL‐17 promotes tumor angiogenesis, proliferation, and invasion.^28^, ^29^ Furthermore, Tregs are generally considered to be immunosuppressive cells.^30^, ^31^, ^32^ Therefore, the roles of CD4^+^ TILs are unclear and further investigation is needed for gastric cancer with peritoneal metastasis.

Several investigations demonstrated the importance of MDSCs in tumor‐associated immune suppression.^33^, ^34^ In this study, only high CD8/CD33 ratio was associated with favorable OS in multivariate analysis. CD8/CD33 ratio implies the infiltrate composed of many CD8+ lymphocytes with low numbers of immunosuppressive CD33^+^ MDSCs, and hence a better situation to fight the tumor growth. We have previously reported that CD33^+^ MDSCs were recruited to peritoneal metastatic lesions via platelet aggregation, which contributed to tumor progression.^35^ It is considered that the accuracy of a prognostic factor was further improved in combination with CD8.

This study had several limitations including the retrospective study design and the use of a small sample size of patients obtained from a single center. It is relatively difficult to obtain a lot of specimens from peritoneal metastatic lesions that have not undergone chemotherapy. Therefore, further prospective multicenter and large‐scale studies are needed to reveal these results and elucidate the significance of the molecular processes involved in TILs infiltration.

## 5 CONCLUSION

High CD8/CD33 ratio and high infiltration of CD8^+^ lymphocytes into peritoneal metastatic lesions were associated with favorable prognosis in gastric cancer patients with peritoneal metastasis. CTLs play an important role in antitumor effect for peritoneal metastasis, although immunological ignorance often occurs in peritoneal cavity. We have to develop the treatment strategy for induction of CTLs infiltration.

## CONFLICT OF INTEREST

The authors declare that they have no competing interests.

## AUTHOR CONTRIBUTIONS

*Conceptualization, investigation, writing‐Original Draft, methodology*, T.Y.; *Investigation, data curation*, J.K. and K.O.; *Visualization, investigation*, H.S.; *Formal Analysis*, M.S.; *Investigation, formal analysis*, S.T.; *Software*, H.M.; *Methodology*, I.M.; *Visualization, methodology*, K.N.; *Formal Analysis*, H.T.; *Supervision*, I.N.; *Writing ‐ Review & Editing, project administration*, S.F.

## ETHICAL STATEMENT

This study was approved by the Kanazawa University Hospital Review Board (Permission number 2789). Written informed consent was obtained from all patients.

## Data Availability

The datasets used and analyzed during the current study are available from the corresponding author on reasonable request.
